# A framework for equitable virtual rehabilitation in the metaverse era: challenges and opportunities

**DOI:** 10.3389/fresc.2023.1241020

**Published:** 2023-08-24

**Authors:** Mirella Veras, David R. Labbé, Joyla Furlano, David Zakus, Derek Rutherford, Barry Pendergast, Dahlia Kairy

**Affiliations:** ^1^ Centre de recherche interdisciplinaire en réadaptation du Montréal métropolitain (CRIR), Montreal, QC, Canada; ^2^ École de technologie supérieure (ÉTS), Université du Québec, Montreal, QC, Canada; ^3^ Atlantic Fellow for Equity in Brain Health, Global Brain Health Institute, Dublin, Ireland; ^4^ Dalla Lana School of Public Health, University of Toronto, Toronto, ON, Canada; ^5^ School of Physiotherapy, Dalhousie University, Halifax, NS, Canada; ^6^ The AGE-WELL Metaverse Working Group, Toronto, ON, Canada; ^7^ École de réadaptation, Faculté de Médecine, Université de Montréal, Montreal, QC, Canada

**Keywords:** metaverse, rehabilitation, framework, digital health, physiotherapy, occupational therapy, virtual reality, telerehabilitation

## Abstract

**Introduction:**

Metaverse technology is spurring a transformation in healthcare and has the potential to cause a disruptive shift in rehabilitation interventions. The technology will surely be a promising field offering new resources to improve clinical outcomes, compliance, sustainability, and patients' interest in rehabilitation. Despite the growing interest in technologies for rehabilitation, various barriers to using digital services may continue to perpetuate a digital divide. This article proposes a framework with five domains and elements to consider when designing and implementing Metaverse-based rehabilitation services to reduce potential inequalities and provide best patient care.

**Methods:**

The framework was developed in two phases and was informed by previous frameworks in digital health, the Metaverse, and health equity. The main elements were extracted and synthesized via consultation with an interdisciplinary team, including a knowledge user.

**Results:**

The proposed framework discusses equity issues relevant to assessing progress in moving toward and implementing the Metaverse in rehabilitation services. The five domains of the framework were identified as equity, health services integration, interoperability, global governance, and humanization.

**Discussion:**

This article is a call for all rehabilitation professionals, along with other important stakeholders, to engage in developing an equitable, decentralized, and sustainable Metaverse service and not just be a spectator as it develops. Challenges and opportunities and their implications for future directions are highlighted.

## 1. Introduction

The Covid-19 pandemic has been a powerful catalyst for the growth and transformation of digital and technology-enabled healthcare solutions (1). Innovative technologies used to fight the spread of the virus have addressed the diverse needs of patients and providers by using digital technologies to enable virtual care (VC), education and research (1, 2). Healthcare delivery has changed, and the digitalization of patients’ records (data captured in electronic records), assessments, monitoring, and treatment are being forever altered (3). The future is arriving at a faster pace than expected in the new digital era. Some experts believe the Metaverse will significantly change the healthcare landscape by engaging clinicians, patients, educators, and researchers in a virtual world for the assessment, prevention, and treatment of patients, as well as education and research (4).

The term Metaverse is everywhere, from computer sciences to economics, medicine, healthcare, education, psychology, architecture, military, fashion, dating, sports, arts, etc. Although it first appeared in 1992 with the science fiction novel *Snow Crash* by Neal Stephenson, the roots of Metaverse date back further in the 20th century (5, 6). The definition of Metaverse can vary in its field of use. Still, in summary, it is the next generation of the internet, where a dynamic, interoperable (or immersive) 3D virtual space enables people to work, shop, study, be physically active, and interact with others by using virtual reality (VR), augmented reality (AR) or mixed reality technology all aided by artificial intelligence (AI) (6). For clinical purposes, we will use the term Clinical Metaverse, defined here as the *Use of an Internet 3D virtual space platform to deliver clinical services that include assessment, prevention, monitoring, interventions, education and consultation*.

VR and AR are relatively recent tools used in rehabilitation. VR has been used in several areas of physiotherapy, occupational therapy, and speech therapy to improve upper limb function in stroke survivors, hand therapy, pain management, rehabilitation from COVID-19, lower back pain, balance treatment, cognition, communication, and acquired brain injury rehabilitation (7–9). AR is used in combination with conventional therapy for the treatment of balance and fall prevention in older adults, lower and upper limb functionality in stroke, phantom pain syndrome, and treatment for gait freezing in those with Parkinson's disease (10, 11). It is generally used to augment the physical environment with cues and visual feedback. VR is often used for treatment immersion, giving full control over the user's environment, also often together with virtual self-avatars.

As proposed in 2007, Metaverse needs four types of technologies to enable the 3D internet experience: (1) Augmentation: technologies that layer information onto our perception of the physical environment; (2) Simulation: technologies that model reality; (3) Intimate: technologies that are focused inwardly on the identity and actions of the individual or object, and (4) External: technologies that are focused outwardly towards the world at large (12). A Metaverse technology taxonomy with four key components has been proposed: AR, Virtual Worlds, Lifelogging and Mirror Worlds. VR and AR were previously described; Lifelogging refers to technologies that allow a user to record or monitor their internal states to augment their lives (e.g., smartwatch to monitor the heart rate of physical activity) and Mirror Worlds to capture and create a virtual simulation of a person's external reality (e.g., Google street view) (12).

Despite the concept of the Metaverse being proposed for decades, a unified architecture still lacks. In their respective works, Duan et al. (13) and Jon Radoff (14) have contributed to shaping the architecture of the metaverse. Duan et al. presented a three-layer architecture consisting of infrastructure, interaction, and ecosystem (13), while Jon Radoff proposed a seven-layer architecture based on the value chain of the expected market, encompassing infrastructure, human interface, decentralization, spatial computing, creator economy, discovery, and experience (14). Fu et al. (15) combined Duan et al. and Radoff's architectures and proposed a concept that aligns with the idea that the Metaverse bridges the physical and virtual worlds, utilizing digital twins to connect and fully realize this interconnected reality. It comprises six crucial components: technologies, hardware platforms, systems, software, applications, and ecosystems (15).

While it is not the purpose of this article to explore each component's specifics, it is important to highlight that the Metaverse relies on two essential resources, hardware and software, which hold potential applications in the field of physical rehabilitation. Hardware encompasses critical components such as chips, display screens, interactive devices, and terminals. On the other hand, software includes operating systems, rendering tools, computing capabilities, memory storage, and file data (15). Proper resource management plays a vital role in ensuring the Metaverse's stability and resource utilization. It enables the dynamic allocation of resources according to changing requirements, creating a stable and orderly environment for the seamless functioning of the Metaverse. Diverse hardware platforms that can be used to access the Metaverse, such as XR devices, sensors, computers, mobile phones, and helmets, as well as software and systems like Windows, iOS, and Android, play integral roles in enabling interactions and computing within the Metaverse.

In the context of physical rehabilitation, VR has gained popularity as a valuable tool for treating various conditions. It has been employed in balance training to enhance balance and mobility for individuals with Parkinson's disease (16), post-total knee arthroplasty rehabilitation (16), and chronic neck pain (17). These interventions typically employ different tools, such as sensors, gloves, or VR headsets, to provide real-time feedback within virtual environments. Advancements in technology now encompass augmented and VR, telerehabilitation, and advanced wearables, expanding the possibilities for remote therapy accessibility. There is important application of VR as a technology in physical rehabilitation, wherein hardware and software components within the Metaverse contribute to its effectiveness and advancements in this area.

Examples of specific devices applicable to the virtual rehabilitation world include the 3D Oculus Rift, one of the pioneers in consumer-based VR headsets developed by Oculus in 2014. This type of headset may be useful in making repetitive rehabilitation exercises more immersive and engaging and can simulate movements used in everyday life for people with various physical and neurological impairments. For instance, patients recovering from stroke can use the Oculus device to virtually practice reaching for objects (18), which may improve their fine motor skills and hand-eye coordination. In patients with Parkinson's disease, a progressive disease that affects the nervous system, the Oculus Rift has also led to improvements in functional performance (19). A more recently developed device is the wearable Teslasuit, a full-body suit designed to monitor human behaviour and improve performance. Using electro muscle stimulation (EMS) and transcutaneous electrical nerve stimulation (TENS), the suit provides physical feedback based on visual simulation (20). The suit's motion capture system allows researchers to monitor physical performance and access internal information related to the physical state of the user (e.g., cardiorespiratory function) (20). For the user themselves, the suit can aid in building muscle memory and improving one's technique related to physical movements (20). While this device is currently being used for research purposes, its clinical utility is apparent.

The rapid digitization of healthcare must be approached cautiously to avoid increasing health inequities. Virtual care (VC) will be compromised if solutions are not developed to address current limitations and barriers. Many studies have pointed out the limitations of poor access to the internet, privacy and security issues, and technological and digital literacy as barriers to VC in several population groups (21–23). Some criticisms of VC argue that online interactions may adversely affect the continuity of care and difficult patient-provider relationships through impersonal interaction, especially for patients with chronic or complex diseases. In addition, VC may also face many legal and regulatory barriers, including significant variations in rules, regulations, provider compensation, and guidelines for practice (3, 21).

This paper embodies the latest and most comprehensive discussion in the field of Metaverse development. Its novelty lies in its selection of the crucial domains within Metaverse development and its effective adaptation to the field of rehabilitation. Particularly, this work stands as the pioneering work in proposing a Metaverse framework tailored specifically for the rehabilitation domain, encompassing various health professions like physical therapy, occupational therapy, and speech therapy, which collectively contribute to the advancement of rehabilitation practices. To the best of our knowledge, no previous effort has been undertaken to establish a framework that facilitates the development of the Metaverse specifically dedicated to systematizing and formalizing the state-of-the-art advancements in Metaverse technology for rehabilitation purposes. Subsequently, this paper aims to present a framework to ensure equitable and accessible rehabilitation services and research using the Metaverse for delivering care or designing research studies.

## 2. Material and methods

Our proposed framework was developed drawing on McMeekin, Wu, Germeni, and Briggs' “Framework development” method (24). They proposed three phases: “*(1) identifying data to inform the methodological framework; (2) developing the methodological framework; and (3) validating, testing and refining the methodological framework*” (24). For the framework, we followed the first two phases, and the third phase will be conducted concomitantly with the expansion of Metaverse technology and incorporating the proposed elements described here.

Development of the proposed Metaverse Equitable Rehabilitation THerapy (MERTH) framework was thus conducted in two phases. The first phase identified evidence from the literature to inform this framework by searching for previous frameworks or guidance pertaining to the equitable aspects of the Metaverse. Our search strategy was based on a review of literature on equity frameworks, supplemented by additional searches for specific terms associated with the Metaverse (governance, decentralization, humanization, and environmental impact). Searches were performed on PubMed, Google Scholar, and grey literature (on official websites of companies engaged in the development of the Metaverse such as Meta (Facebook Metaverse Company), Microsoft, Descentraland (Metaverse Platform), and NVIDIA) and Metaverse Standards Forum. The main studies used for this first phase were: The Health Equity Framework by Peterson et al. (2021) (25), A Framework for Digital Health Equity by Richardson et al. (2022) (26) and relevant information from The Metaverse Standards Forum (27). These resources were chosen and included because they are highly influential in their fields, as demonstrated by their publication metrics.

The second phase consisted of developing a framework by extracting and synthesizing the main elements of equity, digital health, and Metaverse frameworks. This involved an interdisciplinary team of clinicians and researchers within the fields of rehabilitation, computer sciences, equity, and global health, as well as a knowledge user. After the synthesis, the results were returned to the study team for refinement. This iterative approach was followed until consensus was reached by the study team on the domains and subdomains to be included.

## 3. Results

### 3.1. Metaverse equitable rehabilitation therapy (MERTH) framework

**Figure 1:**
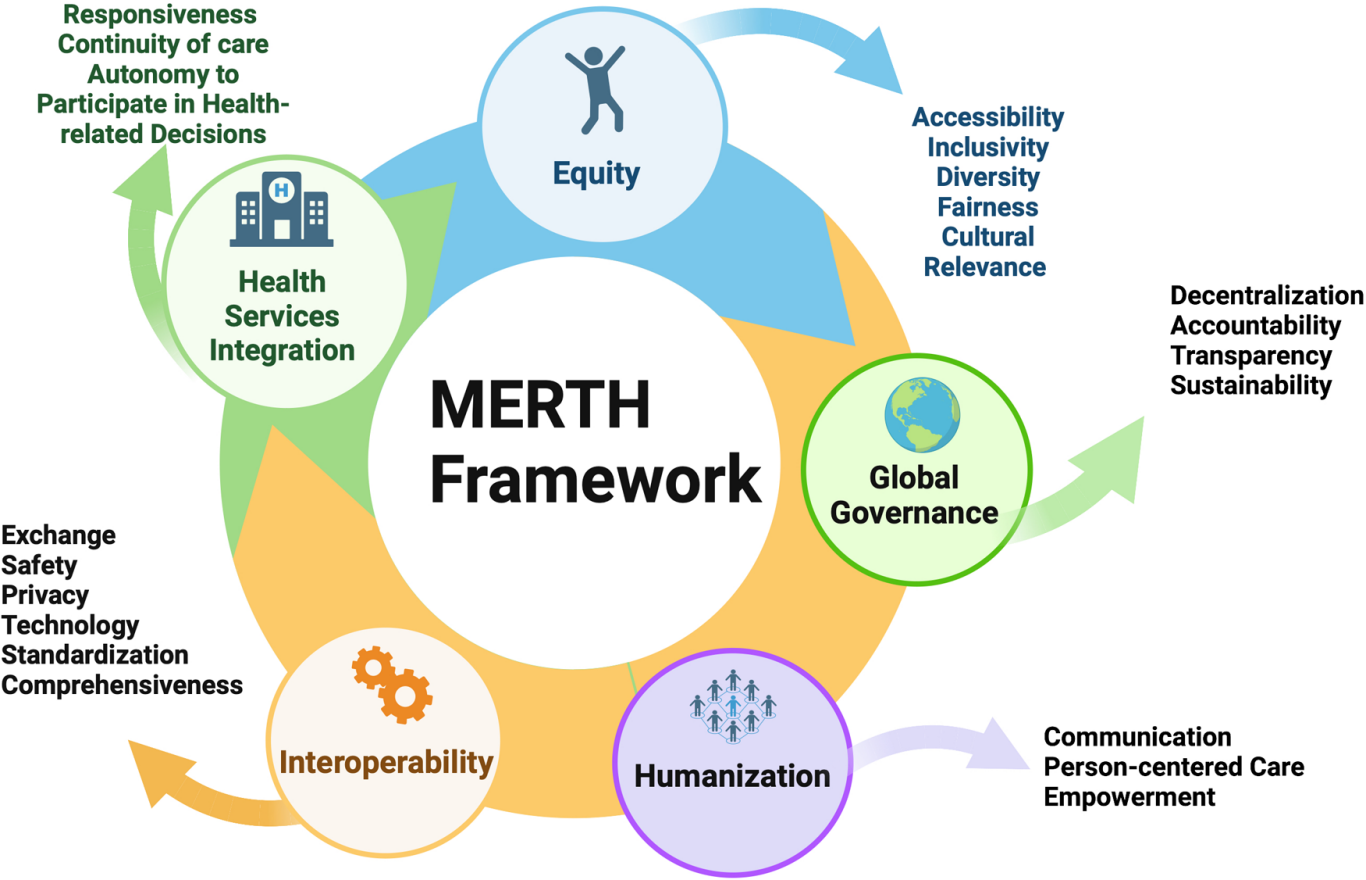
Metaverse equitable rehabilitation therapy (MERTH) framework. Figure 1 was created with BioRender.com.

The proposed framework is composed of five domains, each with several subdomains, to guide and support both researchers and rehabilitation clinicians who use the Metaverse, as outlined in Figure 1.

#### 3.1.1. Equity (accessibility, inclusivity, diversity, fairness, cultural relevance)

Health equity is a priority in public health and also in rehabilitation services (25). Canada, like other countries, has a growing aging population, and 22% of Canadians have at least one disability (28, 29). These factors significantly impact the healthcare system and indicate the need for quality rehabilitation services. The Metaverse has the potential to reach everyone around the world, and inclusion, diversity, fairness, and cultural relevance are crucial. There is a need to ensure everyone can access very fast wireless connections and equipment to access VR, such as glasses and headsets. Implementing the Metaverse should include education in digital literacy and system adaptations for people with vision, hearing, and physical impairments. For example, a person with blindness or low vision needs to have a computer camera capable of capturing head and hand movements.

Haptics and haptic technology are crucial to those deafblind individuals who depend on touch to interact with the world and present a revolutionary intervention to improve accessibility. Haptics technology facilitates interaction with tools and other users by transmitting the sense of touch over distance (30). It enables the exploration of communication between clinicians and patients using immersive VR experiences facilitated by full-body suits equipped with haptic feedback sensors and sensors responsible for real-time transmission of stimuli that accurately convey force, vibration, and range of motion, resulting in a highly immersive interaction (30). Many studies have reported that adding haptic interfaces to provide feedback to patients facilitates interaction with digital objects and enhances the patient's sense of presence within the virtual rehabilitation environment (30). People with severe motor or hearing disabilities as well as those with a combination of impairments or who are deafblind must have accessible Metaverse options to facilitate communication through hand gestures and other resources specifically designed for non-verbal users.

Further, to increase the accessibility of Metaverse technology in rural and remote areas, the development of “Metaverse cafes” where Metaverse technology such as headsets, interaction and sensory feedback devices, and sufficiently fast internet connection could be publicly accessed would be beneficial. These would allow communities to have greater public access to this technology and would minimize the number of technological resources needed as they could be shared among community members. However, there would be other considerations with this, such as ensuring the sustainability of these cafes and providing transportation support to community members living in these remote areas.

As we strive to enhance accessibility, it is essential to guarantee that the services maintain their quality and effectiveness. In a recent randomized controlled trial investigating the therapeutic effects of Metaverse rehabilitation for cerebral palsy, researchers compared the impact of Metaverse physical therapy and conventional physical therapy on different outcomes (including gross motor function, cardiopulmonary function, activities of daily living, quality of life, and the perceived risk of COVID-19 transmission) (31). The study's findings revealed a significant difference in the improvement of gross motor function measures between the two types of therapy. Specifically, the group undergoing Metaverse physical therapy showed notable advancements in their gross motor function compared to those receiving conventional physical therapy, further supported by the outcomes of similar research in the field of VR rehabilitation by Arnoni et al. in 2019 (32). In their research, Arnoni and colleagues observed a 1.45%–23.32% increase in gait parameters scores after implementing VR rehabilitation compared to conventional physical therapy (32). These findings emphasize the potential benefits of incorporating Metaverse-based approaches into therapeutic interventions for cerebral palsy patients.

Further, the utilization of VR in managing patients with low back and neck pain showed promising results in a recent study. Significant improvements were observed, including reduced pain levels, faster response time in arm movements, and increased range of motion for neck rotation, session accuracy, speed, and memorization capabilities (33). Moreover, disability decreasing from non-specific low back pain significantly decreased by 17.8% (*p* < 0.001) as measured by the Modified Oswestry Low Back Pain Disability Index, and Neck Disability Index improved by 23.2% (*p* = 0.02). These findings highlight the potential benefits of incorporating VR in pain management and rehabilitation, offering a comprehensive approach to address various aspects of patients' pain (33). Additionally, the study demonstrated promising outcomes concerning adverse events. Participants did not report any deterioration in their condition during the course of treatment using VR in the Metaverse. This favorable result emphasizes the safety and potential of VR as a safe approach to managing patients with low back and neck pain, further reinforcing its value as a therapeutic tool in the field of physical rehabilitation (33).

It becomes even more imperative to ensure a healthcare system that aims for justice strives to provide equitable and unbiased access to health services for all individuals, regardless of their background, socioeconomic status, or other personal characteristics. Fairness demands that patients have equitable access to rehabilitation services as needed. Patients with and without private insurance need access to all required Metaverse equipment for their assessment or treatment. Health insurance plans should cover treatment and equipment costs. Additionally, public health systems should provide alternative access to this technology to those who need it and may not be covered by insurance. The current health system raises questions about the fairness of patient inclusion (28). Non-clinical factors such as the burden on rehabilitation resources, the age of the patient, and the ability to learn are some factors that can lead to bias and, consequently, unfairness in accessing rehabilitation services (34). Most of the time, rehabilitation patients are unaware of the factors determining whether they were accepted or rejected in a rehabilitation program (34).

Another consideration is the discharge of the patient following care, which sometimes occurs without the patient's agreement. A longitudinal observational study measured outcomes of stroke rehabilitation discharge at 6, 12 and 24 months and demonstrated that adherence to cardiovascular risk recommendations and physical activity decreased over time (35). Another study evaluated the effectiveness of additional weekend physiotherapy on mobility impairments in high-risk older adult patients admitted to an acute medical unit. Findings show that further physiotherapy during the weekend significantly improved patients' ability to complete activities of daily living (36). Metaverse has the potential to provide individuals with enhanced treatment and more frequent follow-ups.

An example of incorporating diversity into rehabilitation services is having Metaverse avatars customized to patients' gender, body types, cultural backgrounds, and other preferences. Patients can have options to choose how to represent themselves. Research indicates that higher levels of avatar identification are associated with increased motivation (37, 38). It is assumed that as individuals perceive a stronger connection between their own identity and the avatar, their motivation to engage in avatar-related tasks is likely to be higher (39, 40). Furthermore, it is suggested that this heightened motivation, resulting from the identification and self-association with the avatar, can potentially enhance performance and recovery (41). It is assumed that as individuals perceive a stronger connection between their own identity and the avatar, their motivation to engage in avatar-related tasks is likely to be higher (39, 40). In immersive VR, when the avatar is viewed from a first-person perspective (42), Waltemate et al. (2018) found that personalized avatars have a considerable impact on body ownership, presence, and dominance when compared to generic avatars, with a similar degree of realism and graphical quality. These findings underscore the significance of personalized avatars that closely resemble users' real-world appearances to enhance user engagement and immersion in VR contexts such as social VR or medical VR-based therapies. For individuals with disabilities in particular, avatar customization to mirror their abilities or assistive devices can foster a sense of inclusivity and representation (43). Zhang et al. (2022) (43) found that a significant number of individuals utilize assistive technologies in their avatars to visually express their disabilities. Although participants often aimed to accurately represent their physical selves, they occasionally opted to omit their disability identity in specific situations or because they wished to highlight other aspects of their identity.

Avatar creators for the clinical Metaverse need to consider race, ethnicity, and other cultural elements when developing avatars, as well as patients' and clinicians' beliefs, values, and preferences. Avatars should exist as a means of self-expression and agency, allowing users to feel empowered in their own clinical care. To achieve this, people from diverse backgrounds should be involved in avatar development to ensure that their perspectives and life experiences are accurately represented. Partnerships with culture-specific organizations and communities, as well as targeted outreach, may be beneficial here. Community members' participation in this process would ensure that avatars are trauma-informed and do not perpetuate harmful stereotypes or reinforce power hierarchies that exist in the real world. Avatar creators should conduct studies to assess cultural sensitivity of avatars, as well as consult experts in equity, diversity, and inclusion to further ensure that possible cultural stereotypes are not promoted.

While the Metaverse provides unique opportunities for intercultural connection via a virtual world environment, it is also necessary to ensure that its technology is culturally relevant; like virtual avatars, virtual environments should be customizable. To do so, it is vital to ensure that Metaverse technology is developed in partnership with diverse and underrepresented communities, especially those living in remote areas, such as Indigenous Peoples. Indigenous populations in Canada, for example, often have unique cultural experiences and needs compared to others. Developing Metaverse technology with these populations means involving them in the brainstorming, creation, and implementation phases to ensure that the technology addresses their needs (e.g., a holistic view of health, history of colonialism and cultural genocide, a deep connection to the land, and community) compared to other populations. In addition, Metaverse technology should be piloted within these communities to ensure that they are appropriate, as we recognize there is cultural diversity across communities even within a single population and cannot assume a “one-size-fits-all” for these technological resources. Features of physical appearance, such as body shape and size, hair color/style, clothing, and accessories that include racial preferences, should have similar costs to most choices.

In addition, rehabilitation in the Metaverse environment has the potential to provide opportunities for rehabilitation professionals to access health data resources and better collaborate with other health professionals, all with the possibility of using artificial intelligence (AI). Opportunities, for example, can include enhanced communication between health professionals and aiding them in performing their clinical tasks such as education, reminder, etc. The landscape of the rehabilitation field is changing with the introduction of AI, defined here as “*one of the fields of computer science with a mathematical process that has the potential to enhance the healthcare system via novel delivery strategies, informed decision making, and facilitation of patient engagement*” (44). AI can reduce therapist workloads and allow them to treat more patients than usual. Rehabilitation professionals must develop data literacy skills to use large datasets and read, analyze, and interpret them as they become single nodes within the clinical network (44).

Furthermore, outside of clinical practice, at an educational level, the Metaverse could also improve training opportunities for health professionals to participate in simulations and VR training exercises to practice their rehabilitation skills (e.g., using specialized equipment and simulating interactions with culturally diverse patients). Rehabilitation in the Metaverse also has the potential to deliver rehabilitation services for long-term risk factor management, consequently improving fairness in the access to rehabilitation services for patients.

#### 3.1.2. Health services integration (responsiveness, continuity of care, autonomy to participate in health-related decisions)

Integration and continuity of care are essential for a health system to provide effective rehabilitation services. Health systems worldwide face challenges in responding to the complexity of the needs of aging populations with chronic diseases (45). Integrated Metaverse rehabilitation services require ensuring different types of services and resources meet the needs of the demographic and health status changes of our communities. Successful integration must ensure that services are offered throughout the person's lifespan and focus across the type of services, providers, and settings, including social support networks and virtual community resources.

When recommending VC, we acknowledge the significance of neuroplasticity, which involves repetition in the process of rehabilitation. Utilizing a virtual world for rehabilitation can offer distinct advantages, such as increased motivation and the ability to facilitate repetitive exercises crucial for promoting neuroplasticity (46). However, we also recognize the importance of caution in spending excessive time “living” in a virtual world. While the immersive nature of the virtual environment can enhance engagement and exercise repetition, it is essential to consider the potential risks associated with prolonged exposure and ensure that proper balance and monitoring are maintained to mitigate any negative impacts. Striking the right equilibrium between maximizing the benefits of virtual rehabilitation for neuroplasticity and mitigating the risks associated with extended virtual immersion is crucial in maximizing the effectiveness and safety of the approach.

Several studies have indicated that adopting a less engaging lifestyle over time can accelerate cognitive decline due to reduced cognitive reserve, impacting the brain's ability to withstand age-related changes and pathology (47). Similarly, emerging evidence suggests that disengaging from the real world in favor of virtual settings may lead to adverse neurocognitive effects. For instance, a recent randomized controlled trial demonstrated significant reductions in the grey matter within the orbitofrontal cortex, a brain region involved in impulse control and decision-making, after engaging in an online role-playing game for 6 weeks (47). Rehabilitation professionals working with virtual environments should be mindful of the risks associated with patients spending more time in a virtual world than in the real world. To mitigate these risks, professionals can include measures to assess the frequency and duration of virtual engagement by collecting data from devices, sensors, or self-report measures, ensuring a balanced approach to rehabilitation in a Metaverse environment (47). Rehabilitation professionals must exercise clinical judgment when deciding whether to recommend rehabilitation in the Metaverse based on their assessments of individuals' mental health, considering factors such as anxiety or depression that may be associated with interactive or virtual environments. Additionally, ethical considerations, such as weighing the potential benefits and potential risks, must be carefully taken into account by clinicians when prescribing any treatment, including those conducted in a virtual environment.

Continuity of care is an essential aspect of quality, safety, and efficiency in care (48). It is understood here as a combination of information (clinical records and information across different settings, providers or levels of care), rehabilitation services continuity across different levels, disciplines, and settings; and the patient-provider relationship (48). Studies have demonstrated that fragmented care management might result in low-quality care with adverse outcomes such as therapeutic errors, avoidable repeated hospitalizations, and increased healthcare costs (49). To address these negative impacts, a care coordinator is fundamental in assisting a patient or a family member/caregiver in navigating the rehabilitation Metaverse to ensure the following elements: professionals working together to reduce the treatment burden; effectiveness of the services; patient involvement in assessing needs; developing a care plan and follow-up; and rapid communication and interaction among clinicians—all of these working together to improve patient satisfaction. This is in line with Pierucci et al.'s definition of care coordination as a “*health system strategy in which many figures are involved, in which the patient should play the leading role together with the collaboration with a care coordinator through self-management, active collaboration and sharing of information*” (49).

The care coordinator's role in a Metaverse clinic is to provide responsive rehabilitation services by ensuring the main response domains are included in the care plan and assisting individuals in navigating in the Metaverse. They can assist individuals in receiving appropriate rehabilitation services and helps ensure that the patient's needs and preferences are met while empowering them. Responsiveness is a multidimensional concept with a human rights lens to achieve a positive health outcome from interventions (50). Fundamental responsiveness domains reported in the literature were adapted to the Metaverse rehabilitation interventions, including autonomy (health-related decisions), choice (meeting with the health provider of one's own choice), communication, confidentiality (privacy), dignity (respectful interaction between patient-provider), and access and quality of care (51). The care coordinator can ensure a care plan to meet the complex care needs of the patient and facilitate the web of care through rehabilitation professionals such as physiotherapists, occupational therapists, speech therapists, and psychologists.

#### 3.1.3. Interoperability (exchange, safety, privacy, technology standardization, comprehensiveness)

Despite the promising potential of the Metaverse in helping solve many of the challenges facing health care delivery, like with other tools for VC, privacy and safety are primary concerns because they depend on technologies, communication infrastructures, collection, transmission, data storage, and remote environments, all of which can be compromised at some point (52). The main goal when designing and implementing Metaverse-based interventions is to address the already identified problems in VC to avoid the Metaverse inheriting existing vulnerabilities and underlying security and privacy challenges. Real-time, fully immersive Metaverse experiences will bring significant data security challenges for interactions between users/avatars and environments and for the interoperability and scalability in the Metaverse (52). All platforms need to follow their countries' personal health information regulations. For example, in Canada and the USA, Clinical Metaverse must follow the requirements of the Key Personal Health Information Protection Act (PHIPA), the Personal Information Protection and Electronic Documents Act (PIPEDA), or the Health Insurance Portability and Accountability Act (HIPAA). These ensure that personal health information is collected, used, stored, and shared in a way that protects the confidentiality of that information and the privacy of individuals and custodians, and that such personal health information is not collected, used, or disclosed more than is reasonably necessary to meet the purpose of it. There is also a possibility that there will be the need for a personal information Act created exclusively for Metaverse users in the future (53). Healthcare professionals should ensure that patients, clients, and long-term care residents know what personal health information they are collecting and how it is stored, exchanged, used, and protected. Additionally, a healthcare professional must take further steps to protect personal health information in case of loss, unauthorized use, or theft (53). Compared to current telemedicine methods (e.g., medical imaging, phone appointments) in which only certain data can be collected and transported, Metaverse-based rehabilitation allows for the collection and storage of a wide range of data, including data such as performance data, physiological data, and kinematic data.

Interoperability is also an essential characteristic of rehabilitation care to guarantee the continuity of care and improve collaboration between health providers and patients access to their health information. When we apply the concept of interoperability in the Metaverse, it is usually defined as the ability to switch between virtual worlds (i.e., different virtual healthcare settings such as hospitals, clinics, communities, etc.) while maintaining experience and assets such as taking your avatar and medical records with you (54). Another vital element for the Metaverse to achieve its full potential is technology standardization to ensure interoperability across systems and platforms. Interventions should be designed to enable patients to seamlessly transfer their avatars, assets, VR glasses, or any associated devices and personal health information across different platforms without encountering compatibility issues or disruptions to their digital or device belongings. The goal is to ensure a smooth and uninterrupted patient experience, allowing them to seamlessly continue their engagement and access their health-related data regardless of the platform or technology used. When integrating other apps and services, rehabilitation professionals must ensure a seamless Metaverse experience for patients. Many organizations are trying to understand and find solutions for this complex standard landscape. A forum was established with more than 2,000 members to develop interoperability standards and discuss possible solutions for the forecasted challenges (55). Rehabilitation professionals need to be involved in these discussions to address their needs and concerns when Metaverse-based intervention delivery is addressed, to ensure truly ubiquitous technology in services.

#### 3.1.4. Global governance (decentralization, accountability, transparency, sustainability)

Governance in the Metaverse implies three elements: accessibility, interoperability, and privacy. Some argue that users rather than executives should be the decision-makers, but who will govern the Metaverse? And what does it mean for rehabilitation? Content governance is complex and defined as “*a system of norms governing the creation and enforcement of content policies, as well as the distribution of powers among entities responsible for each task*” (56). Insights gained from the past experiences with the Internet and VC have highlighted the importance of building a robust content governance system in order to ensure safety, security, trust, and inclusivity. Will the Metaverse follow local regulations and change accordingly?

For example, if a physiotherapist in Nova Scotia is assessing a patient in the Metaverse, will the physiotherapist follow only the regulations from their province/country? Can the physiotherapist recommend smart glasses or a VR headset approved by the Food and Drug Administration (FDA) but not supported yet by Health Canada? Will the regulatory agencies worldwide work together to regulate devices/technologies used in the Metaverse? There are more questions than answers when considering the global perspective. The Metaverse has the potential to remove many of the geographic boundaries and improve access to rehabilitation services worldwide, but therein lies a problem when considering different health regulations, including those for professionals in other provinces, states, and countries. Should the Metaverse follow the same real-world laws as a digital version of the analog (real) world? Which laws will be different, or which ones will extend to the healthcare Metaverse? These are examples of foreseen issues to be discussed and evaluated from equitable, accountability and ethical perspectives. According to Fernandez and Hui, the Metaverse should be inclusive and outside of local regulations that diminish users' freedom (57). Therefore, rehabilitation professionals should be ready to discuss the international level of legislation, rules, and societal norms, professional responsibility, protection of the public, qualification verification for application to rehabilitation services delivery through the Metaverse and the advantages and disadvantages of a unique international regulation. There is also a need for Metaverse use to be included in accreditation programs, some of which are national and international, if they exist at all in many countries.

Most publications on the Metaverse advocate for a decentralized Metaverse using blockchain technology and a distributed network with a decentralized ownership structure where no single entity can own a Metaverse (58). The ownership is divided by stakeholders, and a more significant number of “holders” results in more decentralization. As part of the governance system, decentralized autonomous organizations (DAO) should focus on increasing diversity to ensure equitable and impactful adoption of rehabilitation services in the Metaverse. DAOs are online communities with a legal structure but no central governing body. DAOs are decentralized and autonomously governed by smart contracts (blockchain technology). As part of the governance system, DAOs must guarantee transparency and accountability and reduce complexity while voting in the decision-making process (59).

Rehabilitation professionals must encourage the participation of underrepresented groups and advocate for their voting rights, their agency and participation. A system of governance must protect populations such as children, older adults, visible minorities, Indigenous peoples, and all people with disabilities and, at the same time, allow innovations. Health technology is advancing, but access needs to be more equitable. As rehabilitation professionals continue to rely on technological innovations to improve patient engagement, access, and outcomes, a new social determinant of health has been identified: the digital divide (22). Although poor internet access and the lack of access to a device to use the technology are the significant impediments causing the digital divide, other factors such as lack of acceptance, motivation, income, race, language and digital skills and literacy contribute to this gap. For example, an older Chinese adult who speaks primarily Mandarin at home is less likely to use the Metaverse for rehabilitation if English is the only language available. Hence, rehabilitation professionals, patients representing diverse population groups, creators, and regulators must be involved with the decision-making process of Metaverse governance (59).

Other important governance questions to consider are: what are the environmental impacts of the Metaverse, and what is the role of rehabilitation professionals regarding the effects of climate change on health? Many experts believe blockchain technology establishes a transparent, immutable, decentralized network for the Metaverse. It operates autonomously with a decentralized content ledger and authorizes several parties to transact between themselves and record those transactions on the blockchain without a centralized authority (60). The technology enables transactions that ensure user integrity, privacy, and reputation and serves as a repository to store data. The huge advantage of blockchain technology for rehabilitation interventions is ensuring data privacy and security to prevent sensitive information leaks to others (60). Further, the technology eliminates the potential for fraud or identity theft issues. Many Metaverse projects incorporate NFTs (Non-Fungible Tokens) to authenticate ownership recorded on the blockchain. NFTs can be used to represent patients and providers with a unique avatar. Sensitive data such as biometric postures and gestures from AR/VR devices, haptic gloves, smart devices, and smart glasses of patients must be acquired to create a digital avatar for rehabilitation interventions. However, data acquisition presents a challenge in guaranteeing high-quality and authentic data due to the long process needed to complete transactions because of the vast amount of data and massive data storage hardware required (60).

While blockchain technology has the potential to make rehabilitation services in the Metaverse more secure and private, it raises climate concerns due to the streaming of data and its analogy to the carbon footprint of computational demands of mining and subsequent substantial contribution to global carbon emissions. These concerns about carbon emissions have been previously mentioned, and many companies such as Google, Amazon, and Microsoft have expressed their commitment to carbon-free energy (61). In the meantime, other alternatives to substitute or improve blockchain technology have been proposed, such as AI, 3D printing, VR, AR, the Internet of Things (IoT), and other features, but they may compromise the decentralization that is one of the crucial pieces of Metaverse governance.

In addition to reducing patient (even therapist) travel for services, especially a concern for remote communities, it is possible that the Metaverse could positively impact the environment over the long term by substantially reducing carbon emissions through the substitution of physical efforts for digital ones, reducing the number of commuting employees and physical plant considerations resulting in fewer transport and provider-related emissions, replacing real-world presence with virtual interactions (62).

The World Health Organization emphasizes that the risks to human health due to climate change undermine many social determinants for good health, such as access to health care and social support structures, equality, etc. (63). In 2019, the Canadian Association of Physicians for the Environment (CAPE) published a toolkit to enable physicians to fulfill the World Organization of Family Doctors (WONCA) goal through its declaration calling on family doctors worldwide to act on planetary health (64, 65). Rehabilitation professionals must understand the environmental impact implications of the Metaverse and work with developers, decision-makers, clients, and professional organizations to limit and mitigate these risks to protect the health field (66) and advocate for a sustainable Metaverse of rehabilitation interventions.

#### 3.1.5. Humanization (communication, person-centered care, empowerment)

Humanization is “*a process of communication and caring among people that leads to self-transformation, an understanding of the fundamental spirit of life, and a sense of compassion and unity*” (67). The term “Humanized Metaverse Rehabilitation” is described here as an approach to make the Metaverse, a digital experience, more similar to real life by providing meaningful, respectful, and participative assessment and treatment, taking into consideration the patient's needs, traumas, emotions, culture, and behaviours. As technology evolves, it is vital to have virtual spaces created to produce human experience and acknowledge our humanity. Technology, in all its enhancement, must continue to be a resource to meet the needs of human beings.

A growing literature discusses the need to humanize healthcare, where humans may develop symptoms of pain, discomfort, panic, anxiety, difficulty breathing, and feel isolated and lonely during their single or multiple healthcare visits to treat life-threatening illnesses or chronic diseases (68, 69). Historically, people with disabilities have faced segregation, discrimination, and dehumanization. Despite great strides over the past decades, some advances in medical technology have raised concerns about new eugenic practices. Experts from The United Nations warned against these practices and “ableism” in clinical approaches towards “eliminating” deemed undesirable human characteristics and the promotion of euthanasia to systematically diminish disabled lives (70). For example, it has been suggested that “death with dignity” implies that a person with a disability or illness “lacks” dignity because of their disability or illness condition and must die to be dignified (49). Some disabilities may result from inadequate access to health care, clinician malpractice due to the assumption about disability, and barriers causing delays or denial of treatment (71).

Rehabilitation within the Metaverse has the potential to enhance social acceptance and solidarity towards disability—and, if it is well designed, enrich human diversity. Humanization of health in rehabilitation encompasses prioritizing a person's care needs while paying attention to his or her physical, emotional, social, spiritual, and intellectual dimensions beyond their clinical needs, considering a person in their entirety (72, 73). The Metaverse has been cited as one of the tools with the potential to change, improve, and possibly transform healthcare, specifically collaborative work, education, clinical care, wellness, research and monetization (74). Clinicians must offer services that integrate humanization or person-centred care to improve patient care quality. Furthermore, Metaverse technology allows real-time monitoring of patients and tracking compliance using wearables, offering a marvelous opportunity to make rehabilitation services better than ever and respond to human needs related to monitoring them. For example, a patient may use body sensors (wearables) and a headset for Metaverse-based rehabilitation treatments, where avatars interact in the virtual world, enabling an immersive experience to control their whole bodies (72).

In this regard, using avatars presents the possibility of humanizing rehabilitation care, and patients and clinicians can use avatars to interact and establish/explore a clinical virtual space. Virtual representations of patients in Metaverse interactions, through avatars, can enable the feeling of being virtually present or virtually co-present, adding synchronous communication (75). Some studies reported that these virtual interactions might help individuals to share personal information and potentially more information than in face-to-face interactions (18, 76). In the virtual space, appearance is equally important as in face-to-face environments (77), and patients can customize their avatars to their personal preferences. To ensure humanization in the clinical Metaverse, patients and clinicians should have the option to choose avatars with a similar physical appearance, should they prefer, and have them behave like actual humans. Avatars, as described in the Equity section, are also a tool for humanization.

The next generation of the Internet needs to work for everyone, and marginalized people should not be negatively affected by unintended consequences of new technologies. Instead, marginalized people should take the lead in shaping it to avoid the Metaverse inheriting the current social media problems such as feelings of exclusion, invisibility, misrepresentation, inadequacy and online bullying (78). To ensure that the Metaverse is inclusive and promotes democracy, design justice and regulation are required. Design justice ensures that marginalized and disadvantaged populations are at the center of the design and that their values, needs, and concerns are taken into consideration to guide the design. National and international laws and colleges of health professionals should regulate users' and clinicians' posts and actions within Metaverse-based rehabilitation services (78).

## 4. Strengths and limitations

To the best of our knowledge, this is the first study to identify domains and subdomains for a clinical rehabilitation Metaverse. Our framework addresses an important gap in the health technology literature by providing a foresighted framework for rehabilitation interventions, education and research using the Metaverse. We have integrated perspectives from different disciplines and knowledge users. Additionally, we proposed a definition for “clinical Metaverse” that can be used for health professionals inside and outside rehabilitation areas and have included grey literature such as blogs, computer sciences internet forums, and video lectures, which have a unique perspective to add information about emerging discussions regarding the Metaverse. The inclusion of this grey literature offers a solution for publication bias by including unpublished information, thereby improving the inclusiveness of different standpoints.

Other limitations need to be acknowledged and addressed, such as selection bias. Although an extensive search was conducted in the PubMed database, computer science area, and grey literature, we may have yet to identify all available data on the topic, and selected frameworks studies were used to inform some domains of our MERTH framework.

## 5. Challenges and opportunities

As a new technological era unfolds, one of the main goals is to overcome persistent problems with current virtual rehabilitation interventions, which have become more prominent with the Covid-19 pandemic. The successful integration of the Metaverse into rehabilitation education, research, and clinical practice depends on the involvement of rehabilitation professionals, health system and technology decision-makers, and patients (including those marginalized) in the Metaverse's development and implementation. While the primary focus of this paper centers on the rehabilitation field of practice, it is worth briefly noting the potential impact of the Metaverse in the field of education. Specifically, the Metaverse is anticipated to bridge the gap between abstract concepts and tangible experiences for rehabilitation professionals. In the field of medicine, discussions have arisen regarding the Metaverse's potential in medical education, emphasizing the importance of incorporating digital health approaches such as virtual reality and the Metaverse in medical and rehabilitation training. To effectively pursue a successful integration of the Metaverse into rehabilitation, we will be called upon to break down the silos and work efficiently and effectively in a shared space with other sectors, where we can collectively address the already known gaps in VC/telerehabilitation that contribute to the digital divide.

The domains and subdomains described in this article are a sine qua non to have accountable, transparent, effective, efficient and inclusive rehabilitation interventions in the Metaverse. The proposed framework can now be evaluated and refined using an intervention or case study to test the recommended domains.
